# Three and Six Months of Ketogenic Diet for Intractable Childhood Epilepsy: A Systematic Review and Meta-Analysis

**DOI:** 10.3389/fneur.2019.00244

**Published:** 2019-03-15

**Authors:** Xian Yu Liu, Jing Chen, Min Zhu, Guo Zheng, Hu Guo, XiaoPeng Lu, Xiaoyu Wang, Xiao Yang

**Affiliations:** Department of Pediatric Neurology, Children's Hospital of Nanjing Medical University, Nanjing, China

**Keywords:** ketogenic diet, intractable epilepsy, childhood, 3 months, 6 months

## Abstract

**Objective:** The ketogenic diet (KD) has been an effective antiepileptic treatment for intractable childhood epilepsy. The appropriate timing to evaluate the effect of KD is 3 months which is still not defined statistically. Therefore, we aim to realistically assess whether spasm remission during the period of 3 months KD can be a prediction index for the therapeutic effect of 6 months treatment.

**Methods:** To investigate the duration and effect of the KD therapy for intractable childhood epilepsy, we searched the relevant articles up to May 20, 2018 from PubMed, Cochrane, Embase, Ovid, Web of Science, Google Scholar, and Conference literature. The inclusion criteria were: (i) confirmed cases of intractable childhood epilepsy, (ii) specific 3 and 6 months follow-up time, (iii) classified spasm remission evaluation. The exclusion criteria were: (i) including other therapy, (ii) unspecific follow-up time, (iii) no specific index for seizure reduction. The data extracted by two researchers independently included proportion of KD duration, seizure remission, year of publication, author, study design, and diet varieties.

**Results:** The search strategy included a total of 542 citations, 18 articles were met the criteria with a total of 1,062 patients included in the final analysis. Compared with 3 and 6 months KD treatment, the rate difference of 50% seizure reduction was −0.01(95% CI: −0.09 to 0.06). And 90% seizure reduction was −0.036(95% CI: −0.090 to 0.017) and seizure free was −0.031(95% CI: −0.081 to 0.020).

**Conclusion:** This meta-analysis provides statistical support that a period of 3 months KD can be a prediction index of 6 months duration in term of spasm remission. The 3 months KD can be implemented to evaluate seizure remission timely and provide personalized early support.

## Introduction

Intractable epilepsy is characterized with “refractory,” “drug-resistant,” “pharmacoresistant” due to the common failure of seizure-control in despite of the monotherapies, the combined therapy with two or more first-line antiepileptic drugs (AEDs) or even an ultimate treatment with maximum tolerated dose (1). Most cases of intractable epilepsy with several appropriate medications still could not be effectively controlled because of the limited effect and inevitable side effects of AEDs (2).

The KD was developed in 1921, which is recommended as an effective option for early treatment of intractable epilepsy with less restrictiveness and side effects. The KD, which advocated low-carbohydrate, adequate-protein, and high-fat diet, has been used to mimic fasting by altering substrate metabolism from carbohydrates to fatty acids and ketone bodies to achieve the antiepileptic effects (3). And the diet has been developed more liberal and palatable with wide application. There are four major kinds of KD at present: classic KD, modified Atkins diet (MAD), medium chain triglyceride diet (MCT), and low glycemic index diet (LGID).

The potential efficacy usually would be evaluated at 3 months (observation stage) since the KD started. However, whether 3 months is the ideal timing to evaluate the remission and forecast later outcome, there is still not a convincing evidence. Therefore, we performed a systematic review and meta-analysis to explore the relation of spasm remission between the 3 and 6 months KD treatment for intractable childhood epilepsy.

## Materials and Methods

### Literature Review for Meta-Analysis

**Figure 1:**
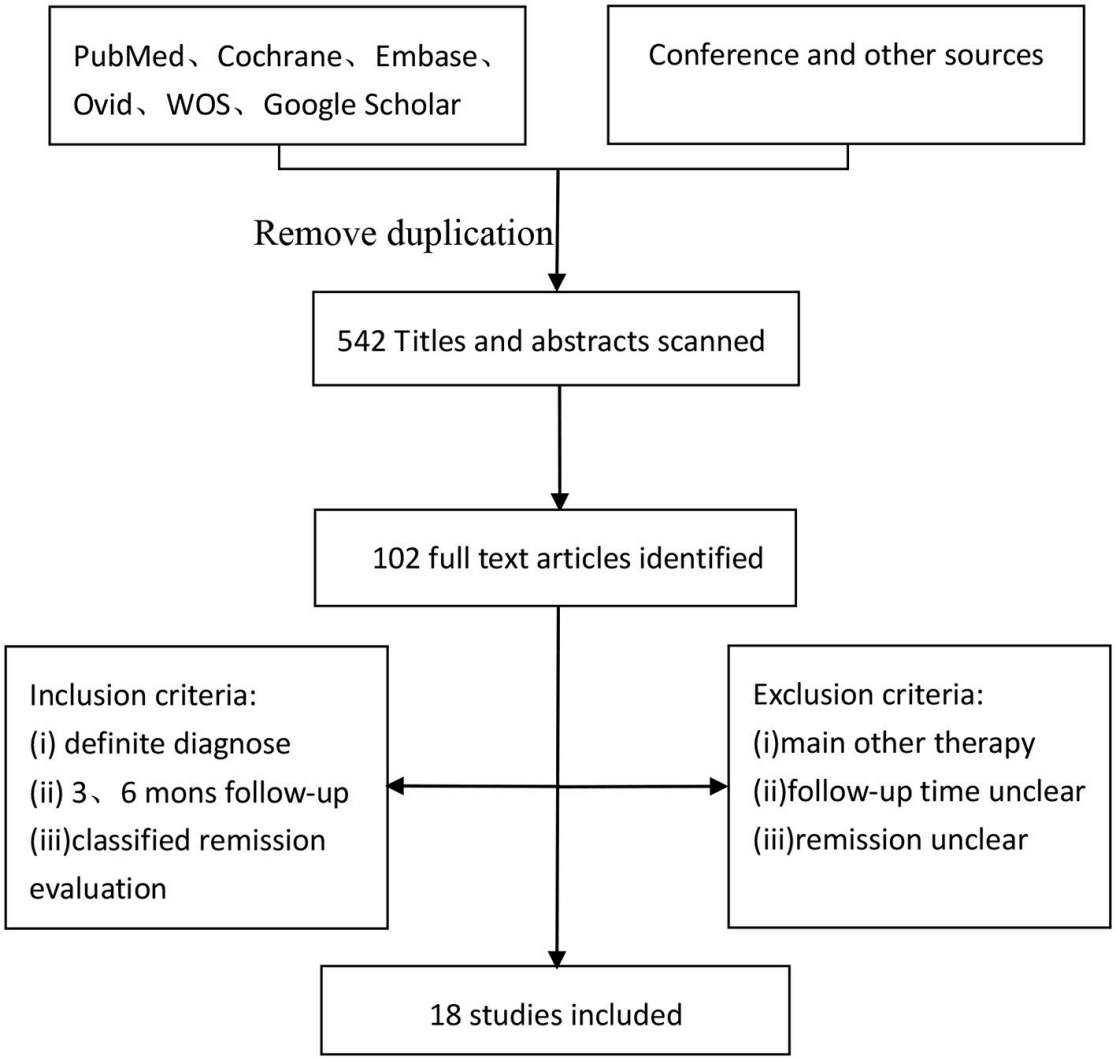
Flow chart of search strategy.

PubMed, Cochrane, Embase, Ovid, Web Of Science (WOS) were searched for the studies using the following key words: ketogenic diet, medium-chain triglyceride, modified Atkins diet, low glycemic index treatment, drug resistant, intractable, medication resistant, drug refractory, and children epilepsy. And each kind of KD also was searched alone with the children epilepsy. Articles were searched up to May 20, 2018. On the other hand, a search of Google Scholar, conference literature and additional sources was conducted to obtain data that may be missed outside of the publication databases (Figure 1).

### Data Extraction

**Figure 2:**
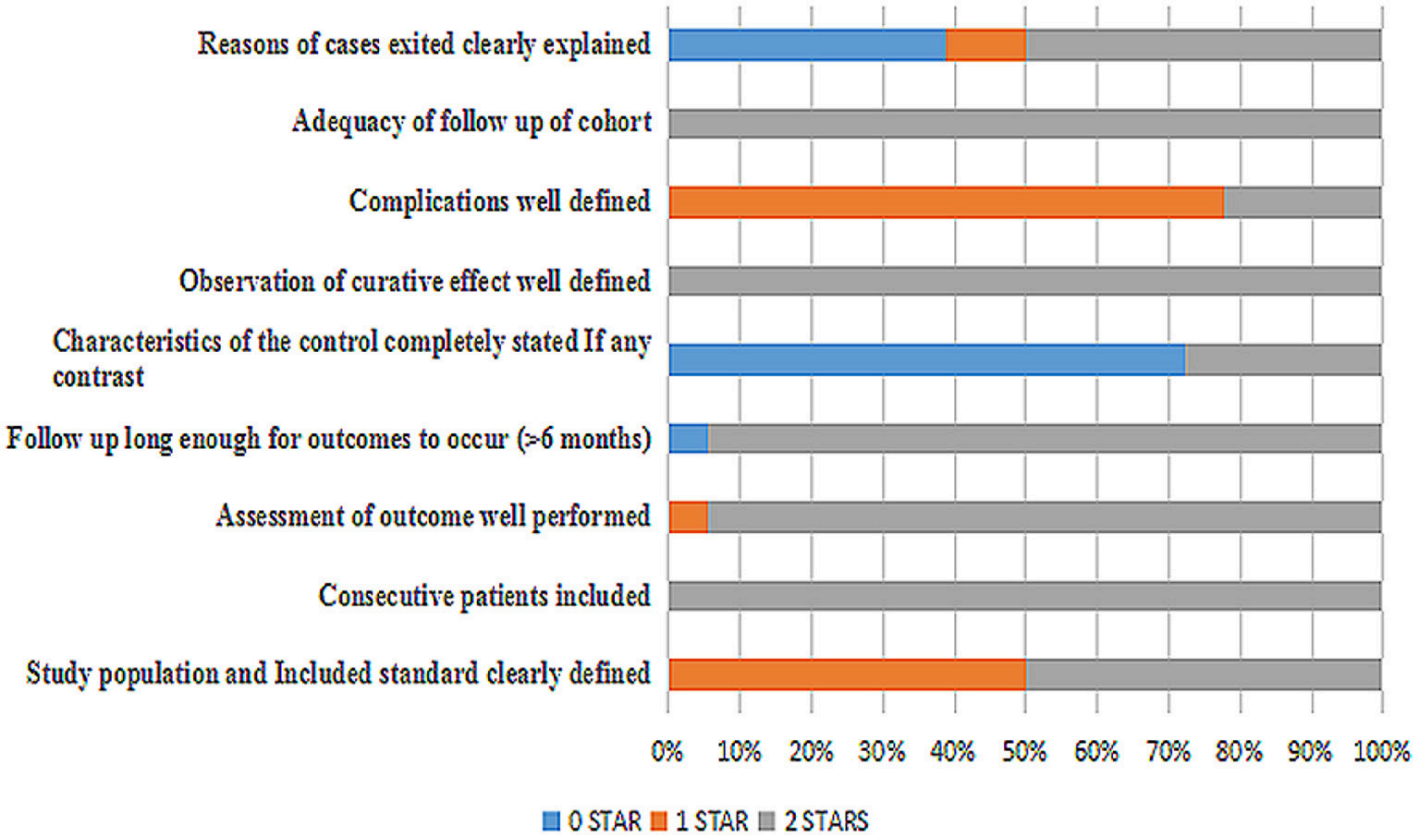
Quality assessment of 18 studies included.

A total of 18 articles (4–21) were met the criteria, including 1,062 patients in the final analysis. For each study included, the full text was retrieved independently by 2 review authors and the following data were extracted: authors, year of publication, seizure remission, study design, diet duration, and diet varieties (**Table 2**). Discrepancies were settled by consensus. In addition, the percentage of seizure reduction was extracted with the corresponding diet duration. Analyses were based on seizure reduction of two time points during diet therapy including classical KD, ketocal milk or ketogenic milk, MCT, and MAD. And quality assessment was conducted by the modified JBI-MAStARI (Figure 2). The following aspects were used: selection of outcome and comparability of study group. Across the studies, most quantitative principle reached 50% or even 100%.

### Data Analyses

Data analyses were conducted using the stataMP 14.0 software. The statistical heterogeneity was assessed by using the rate difference and the *I*^2^ index. A random-effect model was used for quantitatively combined analysis on included studies about 3 and 6 months KD treatment. The efficacy rate was calculated on the numbers of initial patients to avoid pseudo. The changes in the percentages of seizure frequency compared to the baseline seizure frequency was calculated. Then, the meta-analysis was made to calculate the independent and combined efficacy rate of different types of epilepsies. Efficacy was rated using the following categories: (1) seizure freedom (100% seizure remission); (2) seizure reduction by 90% or more (90–99% reduction in seizure frequency); (3) seizure reduction by 50% or more (50–89% reduction in seizure frequency); (4) seizure reduction below 50% (1–49% reduction in seizure frequency and seizure worsened. We planned to work out the rate difference (ES) and 95% confidence intervals (CIs) of seizure freedom, 90% seizure reduction and 50% seizure reduction through a 3–6 months diet treatment. Publication bias was analyzed by Egger's plot.

### Search Results

The incipient search yielded 542 articles. Through screening title and abstract, 102 full-text articles were identified and 18 studies were included. Conference and other sources were searched as the supplement, no articles did not meet the criterions. Figure 1 shows a flow chart for selection of studies. Strict inclusion and exclusion criteria were used in those studies. A total of 1,062 children with intractable epilepsy epilepsies and specific epilepsy syndromes such as infantile spasm, Doose syndrome and Lennox-Gastaut syndrome experienced KD treatment of at least 6 months baseline. Additionally, KD was used in 10 studies (*n* = 665 patients); the MAD and KD was used in 3 (*n* = 93 patients); the MCD and KD was used in 2 (*n* = 193 patients); the ketogenic milk and KD was used in 2 (*n* = 81 patients); and the MAD was used in one (*n* = 30 patients). Table 1 shows characteristics of included studies and Figure 2 shows quality assessment of 18 studies included.

**Table 1:** Characteristics of included studies.

**Study**	**References**	**Classification**	**Prospective/Retrospective**	**Sample size number**	**Male**	**Study design**	**Diet varieties**
1	Vehmeijer et al. (4)	Intractable epilepsy	Retrospective	59	38	Case-control	KD
2	Porta et al. (5)	Intractable epilepsy	Retrospective	27	15	Case-control	KD, MAD
3	Miranda et al. (6)	Intractable epilepsy	Prospective	33	15	Cohort	KD, MAD
4	Amari et al. (7)	Intractable epilepsy	Prospective	33	15	Case-control	KD, MAD
5	Pires et al. (8)	Intractable epilepsy	Prospective	17	1	Cohort	KD
6	Freeman et al. (9)	Intractable epilepsy	Prospective	150	85	Cohort	KD
7	Lambrechts et al. (10)	Intractable epilepsy	Prospective	48	32	Cohort	MCT, KD
8	Neal et al. (11)	Intractable epilepsy	Prospective	145	76	Case-control	MCT, KD
9	Zhu et al. (12)	Intractable epilepsy	Prospective	42	26	Cohort	KD
10	Coppola et al. (13)	Intractable epilepsy	Prospective	38	22	Cohort	KD, ketocal milk
11	Wu et al. (14)	Intractable epilepsy	Prospective	87	62	Cohort	KD
12	Maydell et al. (15)	Intractable epilepsy	Retrospective	143	87	Case-control	KD
13	Eun et al. (16)	Infantile spasm	Retrospective	43	24	Cohort	KD, ketogenic milk
14	Kossoff et al. (17)	Infantile spasm	Retrospective	23	17	Cohort	KD
15	Numis et al. (18)	Infantile spasm	Retrospective	26	15	Cohort	KD
16	Wiemer-Kruel et al. (19)	Doose syndrome	Retrospective	30	24	Cohort	MAD
17	Zhang et al. (20)	Lennox Gastaut syndrome	Retrospective	47	30	Cohort	KD
18	Lemmon et al. (21)	Lennox Gastaut syndrome	Retrospective	71	41	Cohort	KD

**Figure 3:**
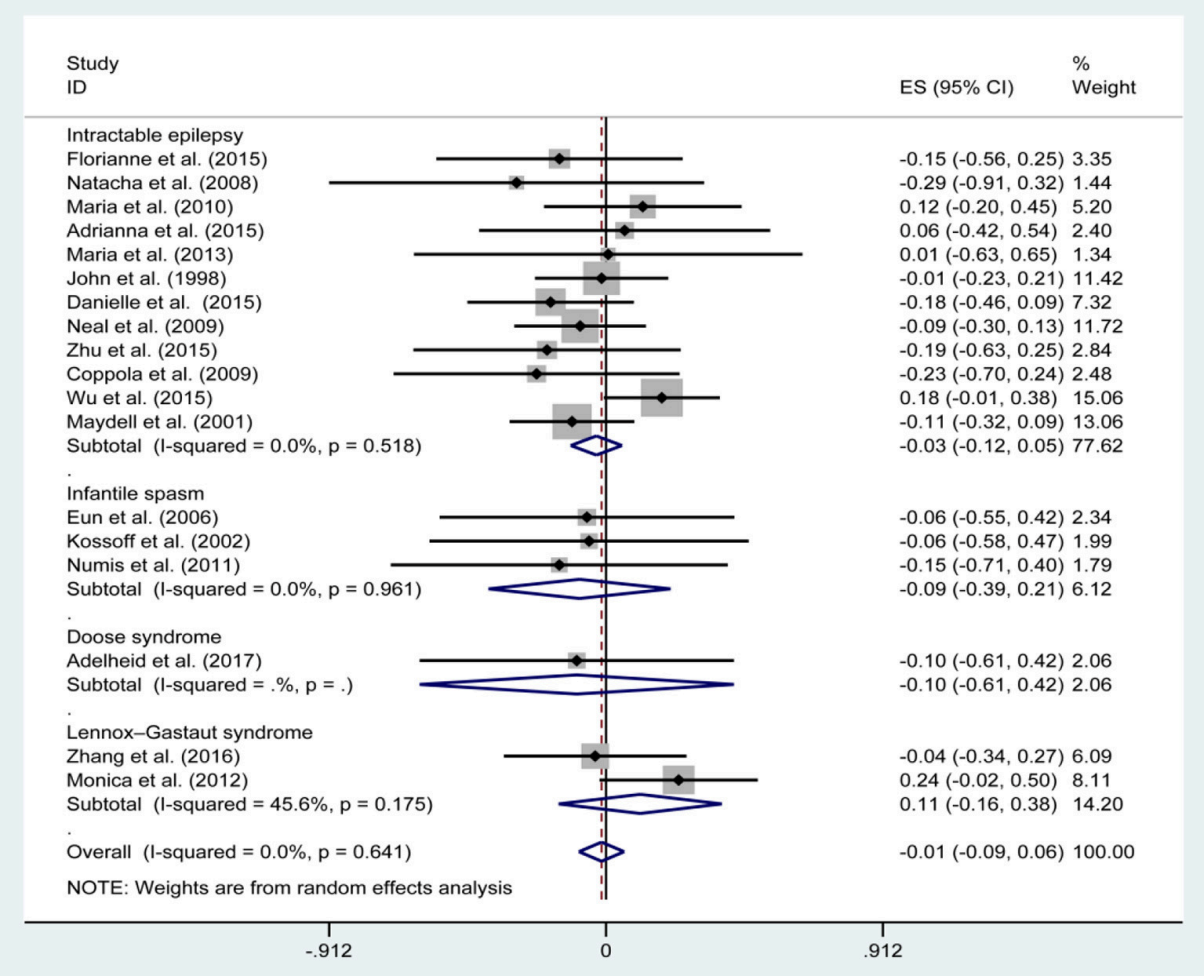
Forrest plot of 50% seizure reduction of 3 and 6 months.

Figures 3, 5, 7, and Table 2 provides the seizure remission of the diet duration, including weighted remission rate difference and 95% CI of the 3 and 6 months treatment in subgroups and total. There was no significant difference between the combined efficacy rates of 50% seizure reduction (*Z* = 0.39, *p* = 0.699). So it is with 90% seizure reduction (*Z* = 1.34, *p* = 0.179) and seizure free (*Z* = 1.19, *p* = 0.233). Compared with 3 and 6 months KD treatment, the rate difference of 50% seizure reduction was −0.01(95% CI: −0.09 to 0.06). And 90% seizure reduction was −0.036(95% CI: −0.090 to 0.017) and seizure free was −0.031(95% CI: −0.081 to 0.020). The meta-analysis revealed the subgroups efficacy rates of the diet still was no significant difference in different epilepsy syndromes such as infantile spasms (West syndrome), Doose syndrome and Lennox Gastaut syndrome.

**Figure 4:**
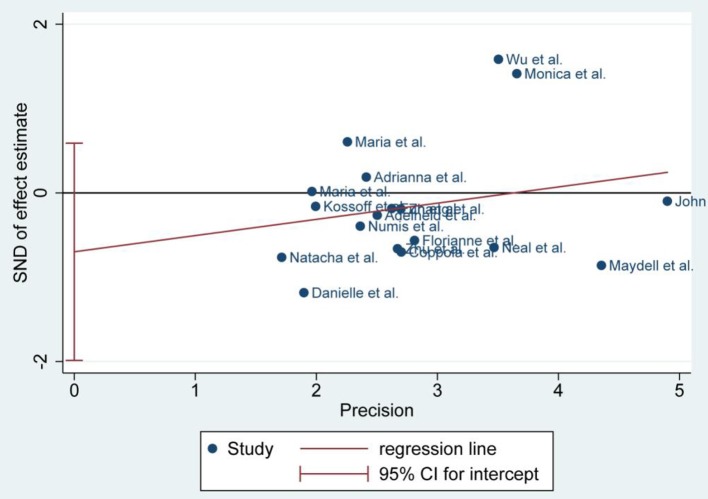
Egger's plot of 50% seizure reduction of 3 and 6 months.

**Figure 5:**
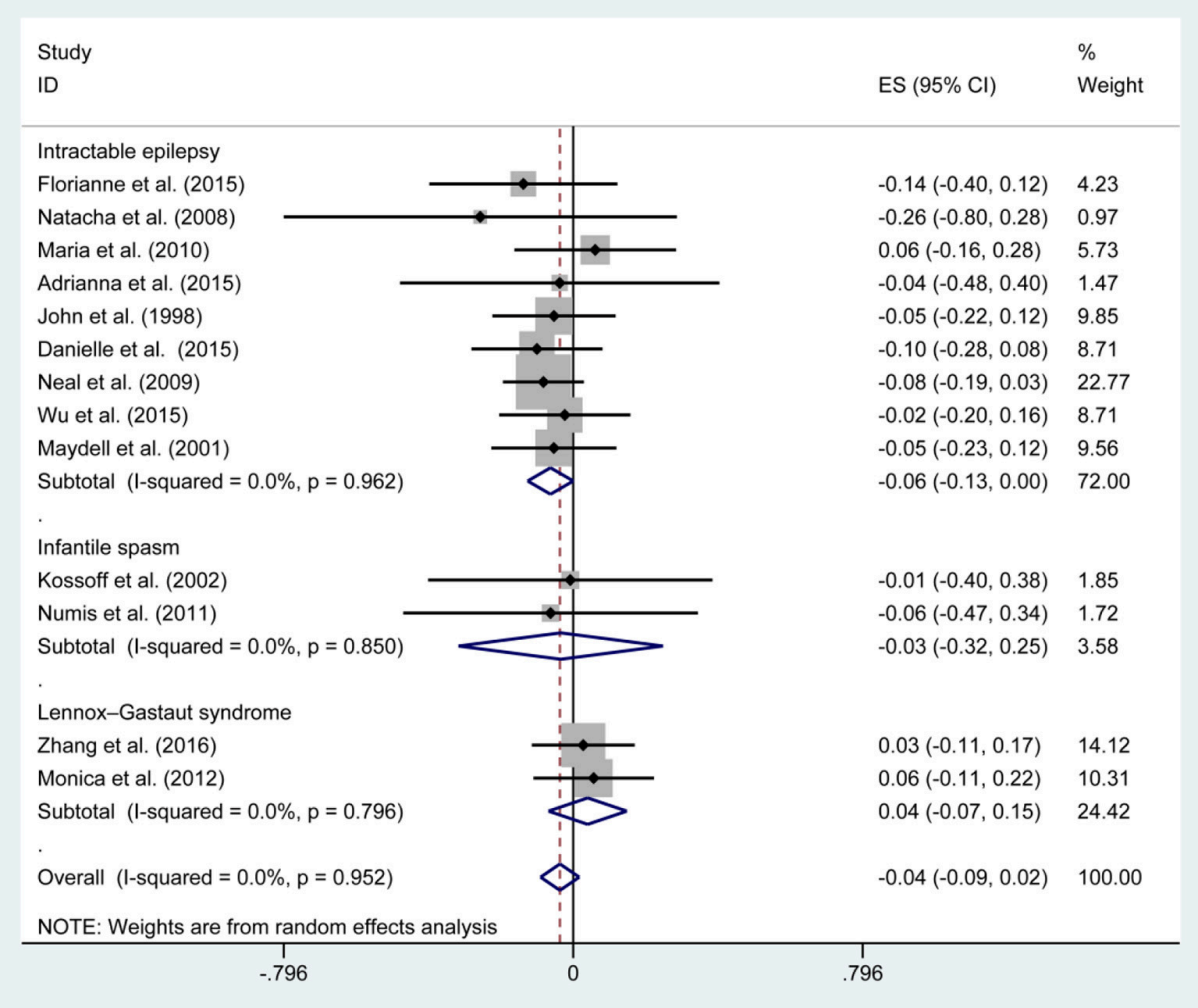
Forrest plot of 90% seizure reduction of 3 and 6 months.

**Table 2:** Analysis results of seizure remission of 3 and 6 months.

**Seizure remission**	**Method**	**Heterogeneity**		**Pooled results**		
		**Q**	***I*^2^**	**ES, 95% (CIs)**	***Z***	***P***
>50%	Rate difference	14.36	0.0%	−0.01(−0.09, 0.06)	0.39	0.699
>90%		5.17	0.0%	−0.036(−0.090, 0.017)	1.34	0.179
Free		19.52	33.4%	−0.031(−0.081, 0.020)	1.19	0.233

### Publication Bias

**Figure 6:**
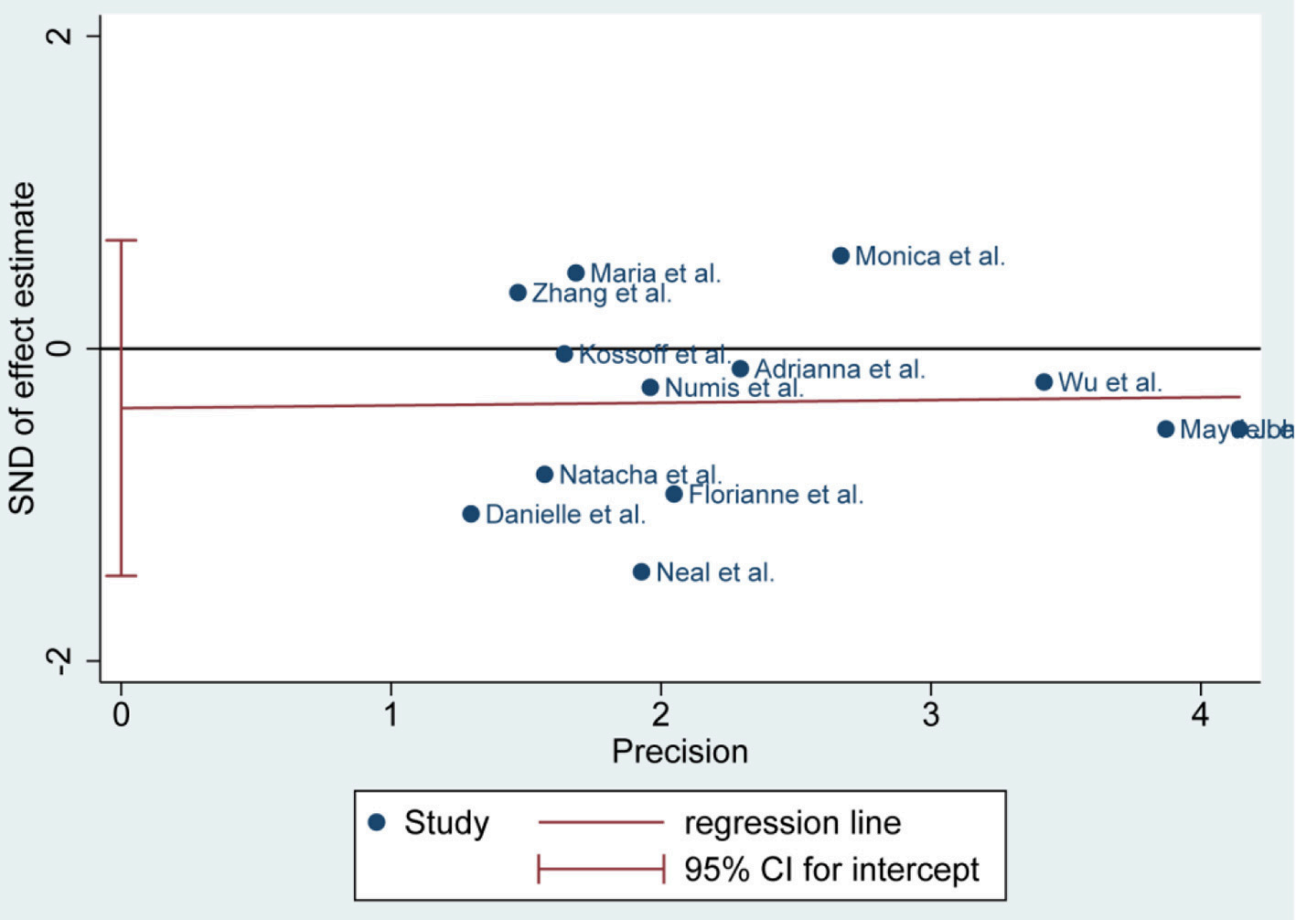
Egger's plot of 90% seizure reduction of 3 and 6 months.

There was no publication bias detected by the Egger's plot (Figures 4, 6 and 8). The Egger's plot revealed a relatively symmetrical distribution of the efficacy rates *at baseline*, which suggested no significant publication bias.

**Figure 7:**
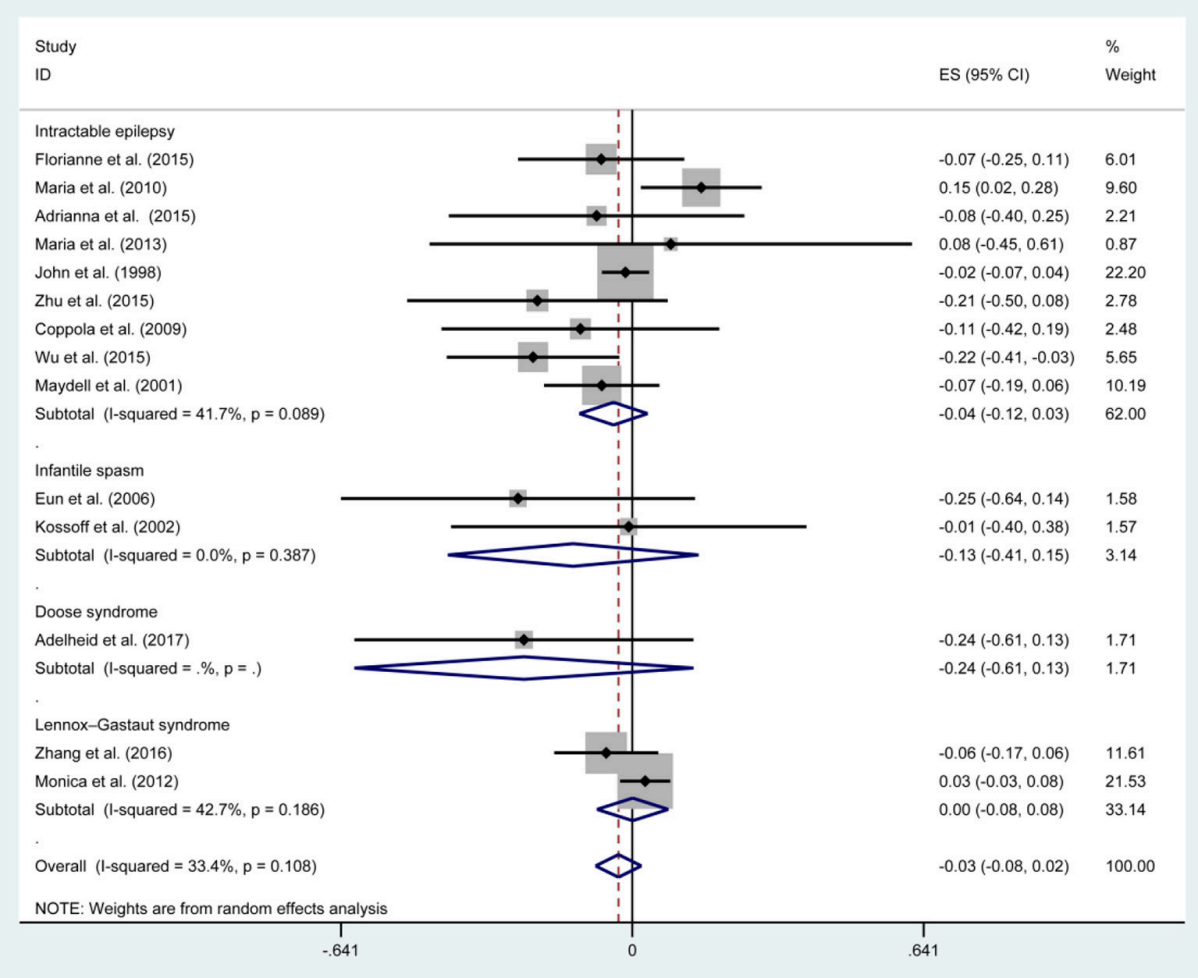
Forrest plot of seizure free of 3 and 6 months.

**Figure 8:**
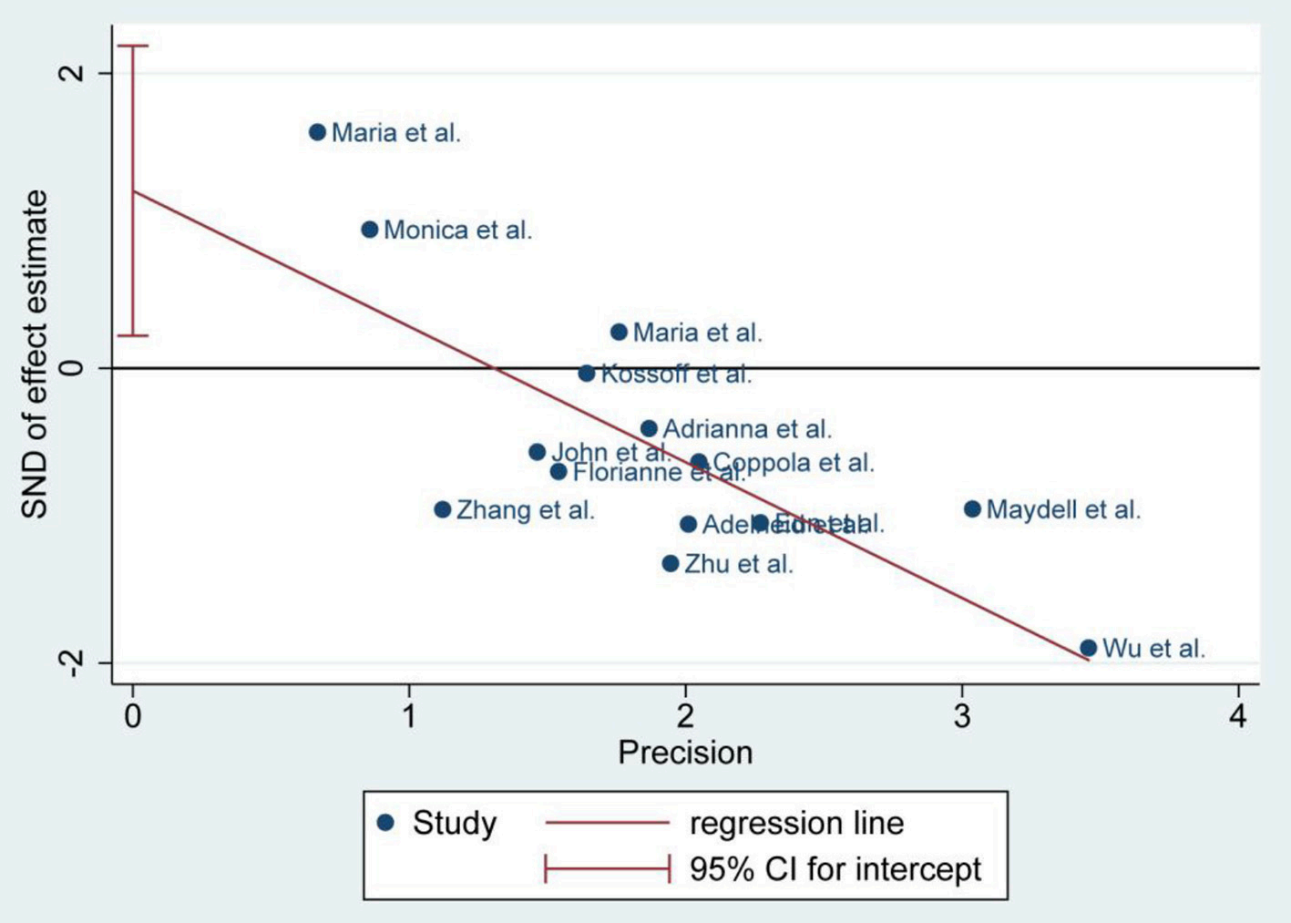
Egger's plot of seizure free of 3 and 6 months.

## Discussion

To the best of our knowledge, our study is the first systematic review and meta-analysis aimed to compare the 3 and 6 months KD efficacy for intractable childhood epilepsy. Even though the food culture around the world was an infinite variety of diets because of the different local conditions and customs, KD had been proven effective to treat children with intractable childhood epilepsy in the present studies. Spasm freedom has been reported in 14–65% of patients within 1–3 months (22).

The meta-analysis was to assess whether a period of 3 months of KD from the seizure remission point of view can be a predictor of 6 months duration to evaluate instant remission and forecast later effect. In our study, the efficacy of 3 months KD treatment did not further increase when the course was extended to 6 months, which favored 3 months as the observation stage for KD therapy. Determine whether KD therapy is appropriate for a patient would be mainly reflected in the reduction of seizure frequency (9). And we found that efficacy of KD treatment at 3 months is significantly related to success at 6 months in providing seizure control. Therefore, 3 months is the ideal observation timing to evaluate the effect of KD through seizure reduction and decide whether to do some fine-tune of anticonvulsant pharmacotherapy.

We evaluated all the relevant studies that focused on the time and efficacy of KD in the children with intractable epilepsy and aimed at providing a comprehensive evaluation of current evidence on the duration of dietary treatment. Different from the previous studies, the first optimum observation stage of 3 months for current effect and next step was compared to 6 months. Moreover, the outcome of efficiency after 3 months duration could be a new dawn for parents to regain confidence through the long treatment time and keep going further (3).

This study supports available evidence that the guiding significance of the 3 month KD treatment referring to seizure remission of more than 50, 90%, and free. However, this study has some limitations. Several studies did not report seizure remission of more than 90% and free separately and therefore we had to exclude some researches.

## Conclusion

This meta-analysis indicates that a period of 3 months of ketogenic diet as a predictor of 6 months duration and 3 months could completely be taken as a course of treatment duration in term of spasm remission. A slight and early modulation should be considered when new-onset or exacerbated adverse reaction generated after 3 months. Further research is necessary to discover the shorter evaluation points and evaluation strategy.

## Author Contributions

XL and JC contributed to the conception of the study, analysis, and manuscript preparation. All authors helped to perform the analysis with constructive discussions.

### Conflict of Interest Statement

The authors declare that the research was conducted in the absence of any commercial or financial relationships that could be construed as a potential conflict of interest.
